# Minimally invasive management of sleeve gastrectomy collection with a nasobiliary drainage tube

**DOI:** 10.1093/jscr/rjad626

**Published:** 2023-11-20

**Authors:** Tejminder S Sidhu, Shaurya Jhamb, Christine Welch, Scott Whiting

**Affiliations:** College of Medicine and Dentistry, James Cook University, James Cook Drive, QLD 4811, Queensland, Australia; Department of Surgery, Townsville University Hospital, 100 Angus Smith Drive, Douglas QLD 4814, Townsville, Queensland, Australia; College of Medicine and Dentistry, James Cook University, James Cook Drive, QLD 4811, Queensland, Australia; Department of Surgery, Townsville University Hospital, 100 Angus Smith Drive, Douglas QLD 4814, Townsville, Queensland, Australia; Gastroenterology North Queensland, Mater Hospital, 25 Fulham Road, Pimlico QLD 4812, Townsville, Queensland, Australia; Department of Surgery, Townsville University Hospital, 100 Angus Smith Drive, Douglas QLD 4814, Townsville, Queensland, Australia; North Queensland Minimal Invasive Surgery (NQMIS), Mater Hospital, 25 Fulham Road, Pimlico QLD 4812, Townsville, Queensland, Australia

**Keywords:** gastric sleeve, bariatric surgery, endoscopic management, sleeve leak

## Abstract

A staple line leak is a feared complication of sleeve gastrectomy. Endoscopic methods have superseded surgical management of small leaks, however large collections often require surgical intervention. Here, we describe endoscopic management of large collection adjacent to the staple line with an 8Fr nasobiliary tube.

## Introduction

Laparoscopic sleeve gastrectomy is one of the most common bariatric procedures in Australia. This procedure provides significant caloric restriction and ghrelin suppression while maintaining an acceptable side effect profile [1, 2]. Complications of the procedure include haemorrhage, abscess formation, stricture, gastro-oesophageal reflux disease, and nutritional deficiencies; however, the most dreaded complication is a staple line leak. Here we present a case of a large staple line collection successfully managed via an endoscopic approach with drainage via a nasobiliary tube placed within the staple line collection and subsequently closed with an over the scope clip.

## Case presentation

**Figure 1:**
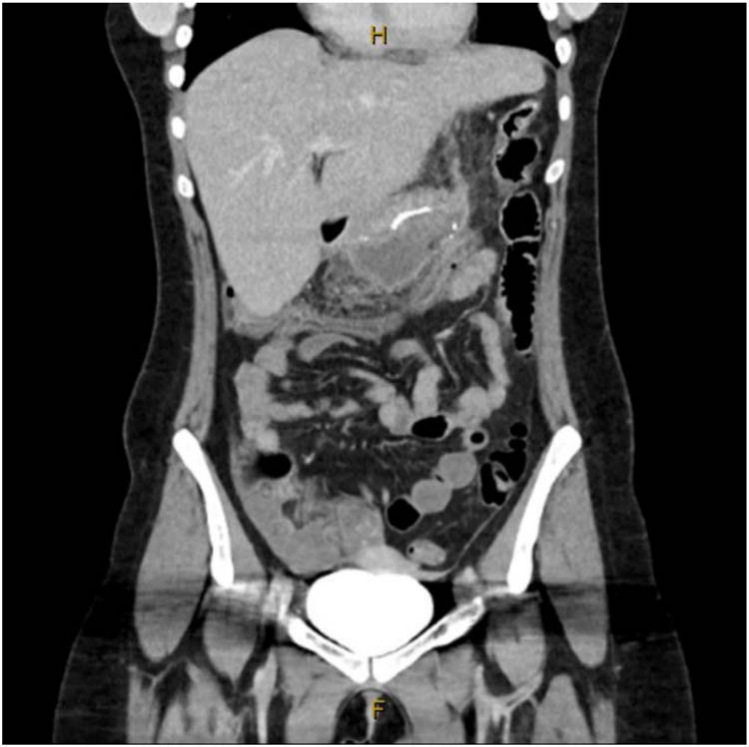
Coronal CT image showing staple line collection at time of presentation. The image shows a 14 × 7 × 5 cm^3^ collection adjacent to the sleeve gastrectomy staple line.

**Figure 2:**
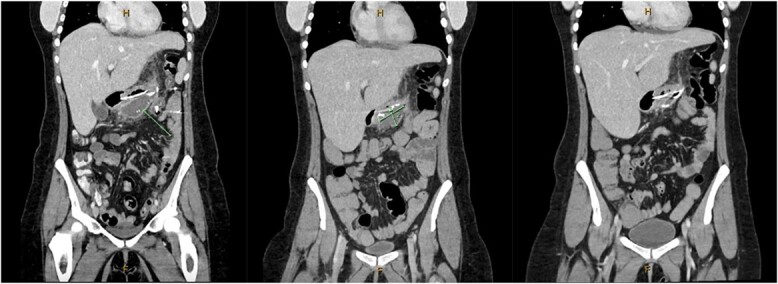
Coronal CT images on Days 2, 9 and 14 post insertion of nasobiliary drainage tube to sleeve gastrectomy staple line collection showing interval reduction in collection size.

A 31-year-old female, 6 weeks post laparoscopic sleeve gastrectomy presented to the emergency department with epigastric pain and vomiting. Initial bloods showed raised inflammatory markers with a white cell count of 15.5 × 10^9^/L and C-reactive protein of 342 mg/L. Initial CT on presentation showed the presence of a 14 × 7 × 5 cm^3^ gas and fluid filled collection adjacent to the staple line (Fig. 1). The patient was resuscitated with adequate fluids and kept nil by mouth. Prompt broad spectrum antibiotics were commenced. An 8Fr firm nasobiliary tube was placed directly into the collection via the defect near the GOJ under endoscopic guidance and placed on suction. During the same endoscopic procedure, a feeding nasojejunal tube was placed to ensure adequate nutrition. Given the extent of the collection, sequential advancement of the nasobiliary tube over the course of 23 days was required to ensure adequate drainage. The collection was monitored with repeat CT scans until resolution (Fig. 2). The GOJ defect was then endoscopically closed with an over the scope clip on Day 23 post presentation. The patient was discharged home post a repeat CT showing complete resolution of the collection and no oral contrast medium extending into the site of the previous collection.

## Discussion

Staple line leaks commonly occur at the proximal portion of the sleeve and are thought to be caused by increased luminal pressure at the angularis incisura and ischaemia caused by dissection and stapling [1, 3]. Staple line leaks are commonly diagnosed on computed tomography (CT). Management of the leak is then commenced, with fluid resuscitation, IV antibiotics and either surgical, percutaneous, or endoscopic intervention. Previously surgical management via laparoscopic washout was the preferred method of management of these leaks, however, in recent times endoscopic management with stenting or clipping has been favoured [4, 5]. Endoscopic management of staple line collections via a double pigtail drain is an established method of dealing with this complication, however, large collections still often require invasive surgical interventions [5, 6].

This case highlights an endoscopic approach to manage large collections post sleeve gastrectomy. The extensive collection in this case was managed in a minimally invasive manner with placement of a nasobiliary drainage tube. Previously described endoscopic management options such as a double pigtail stent would not be able to adequately drain the collection in this case [5, 6].^.^ This novel use of a firm nasobiliary tube when placed on wall suction with its associated length facilitated rapid resolution of this large collection allowing expedient closure of the defect with an over the scope clip.

Other important considerations in management of patients with leaks is nutrition. In this case, a nasojejunal feeding tube was placed to allow enteric feeding throughout. This allowed the patient to avoid total parental nutrition and associated complications [7].

The use of a nasobiliary tube in the fashion described above is not without issues as it requires the patient to remain in hospital connected to wall suction or a vacuum machine whilst tolerating two nasal tubes (one for drainage and one for feeding) in situ until resolution. Appropriate attention needs to be paid to pressure area cares and patient comfort.

## Conclusion

Large sleeve gastrectomy collections can be managed with a nasobiliary drainage tube placed into the collection under endoscopic guidance avoiding the need for invasive surgical intervention.
